# In silico design and synthesis of hesperitin derivatives as new xanthine oxidase inhibitors

**DOI:** 10.1186/s13065-019-0571-1

**Published:** 2019-04-16

**Authors:** Neelam Malik, Priyanka Dhiman, Anurag Khatkar

**Affiliations:** 1 Department of Pharmaceutical Sciences, M.D. University, Rohtak, 124001 India; 2 Laboratory for Preservation Technology and Enzyme Inhibition Studies, Department of Pharmaceutical Sciences, M.D. University, Rohtak, Haryana India

**Keywords:** Hesperitin, Xanthine oxidase, Molecular docking, Antioxidant

## Abstract

**Background:**

Hesperitin, a naturally occurring flavonoid was hybridized with phenolic acids to evaluate its potential to inhibit the activity of xanthine oxidase (XO), a key enzyme which catalyses xanthine to uric acid which is found to be associated with gout and many life style related disorders.

**Objective:**

To develop new xanthine oxidase inhibitors from natural constituents along with antioxidant potential.

**Method:**

In this report, we designed and synthesized hesperitin derivatives hybridized with natural phenolic acids to form ester linkage with the help of molecular docking. The synthesized compounds were evaluated for their antioxidant and xanthine oxidase inhibitory potential.

**Results:**

The in vitro xanthine oxidase inhibitory activity and enzyme kinetics studies showed that hesperitin derivatives displayed a potential inhibition against XO in competitive manner with IC_50_ value ranging from 9.0 to 23.15 µM and HET4 was revealed as most active derivative. Molecular simulation revealed that new hesperitin derivatives interacted with the amino acid residues SER1080, PHE798, GLN1194, ARG912, THR1083, ALA1078 and MET1038 located within the active cavity of XO. Results of antioxidant activity revealed that all the derivatives showed very good antioxidant potential.

**Conclusion:**

Taking advantage of molecular docking, this hybridization of two natural constituent could lead to desirable xanthine oxidase inhibitors with improved activity.

## Introduction

Contemporary boosts in the prevalence of lifestyle-related health disorders results in triggering of dreadful social health disorders. Gout has recently been renowned as lifestyle-related disorders, even though it was historically denoted as ‘the disease of kings’ [1, 2]. The number of sufferers with gout and related painful conditions associated with hyperuricemia in Japan has been significantly amplified during the past 20 years as a consequence of diet alteration along with the accelerated prevalence of metabolic health disorders [3–6]. Gout is an inflammatory disorder which is attributed to elevated amounts and deposition of crystals in the form of uric acid in serum. Uric acid is the end product generated by the catabolism of purine in humans, which is generated by the oxidation of xanthine and hypoxanthine by integral enzyme, xanthine oxidase (XO). For that reason, potent inhibitors of XO are considered to be excellent candidates for curing gout. Considerable accumulation of serum uric acid appeared to be linked to metabolic syndrome [7–10]. Observing the fact that, increase in the level of serum uric acid level results in insulin resistance, hyperuricemia has been thought to be associated with metabolic syndrome as well as type II diabetes. Additionally, rise in serum uric acid in body is typically associated with the progression of hypertension and renal disorders. As a result, hyperuricemia turns out to be the root cause of life-threatening health disorders; the need arises for the substantially more diverse treatment alternative [11–15]. Apart from the multiple adverse reactions, the purine derivative allopurinol and its proactive metabolite, oxypurinol, have long been treated as magnificent preferences for remedial XO inhibition. Keeping in mind the adverse effects produced by purine XO inhibitors; significant efforts were made to explore new, non-purine type XO inhibitors. Febuxostat, identified as non-purine inhibitor of the xanthine oxidase, is likely to be the most significant example to point out because of its current approval by the FDA [16–22]. An alternate potent category of non-purine kind XO inhibitors is the flavonoids and coumarins, the majority of which have already been identified as effective inhibitors of the enzyme. Furthermore, this enzyme-inhibiting activity is in addition correlated with their renowned anti-oxidant activity in vivo, essentially leading to the established and principally beneficial role of flavonoids in human health. A huge frame of scientific literature on flavonoids as XO inhibitors continues to be reproduced. The paramount system to confer a proper constancy for flavonoids without losing the activity was undertaken. Particularly, the research was focused on the targeted flavonoids designed for preventing the disorders associated with xanthine oxidase. It has been now renowned fact that free radicals play an important role in the oxidation reactions, which is the key source of currently prevalent life-threatening disorders. Hence, derivatization of hesperitin, a flavonoids well known for its antioxidant properties and could be applied as treatment remedy for life-style related diseases was done with phenolic acids [23–30].

**Fig. 1:**
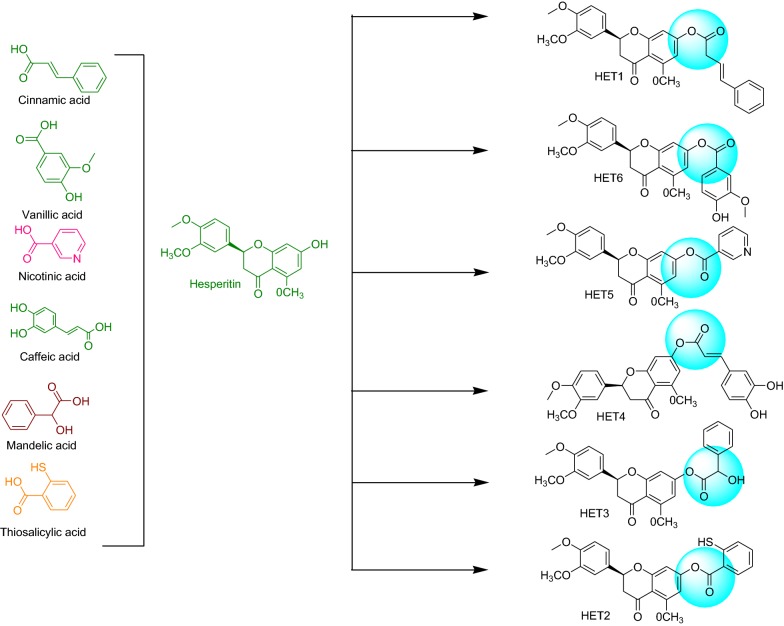
Design strategy of hesperitin derivatives

In our previous work, molecular mechanistic approach was used to find out potentially active natural constituents from different categories of secondary plant metabolites against xanthine oxidase [31]. The outcomes of the study revealed Hesperitin as one of the most active molecules targeted against xanthine oxidase. These results enforced us to develop more potent derivatives of hesperitin by chemical modification that could be served to treat the disorders caused by XO (Fig. 1).

## Experimental

### Chemicals and instrumentation

For this research, the analytical grade chemicals necessary for synthesis and antioxidant activity were purchased from Hi-media Laboratories. The in vitro evaluation of the human xanthine oxidase inhibitory activity was performed by measuring hydrogen peroxide (H_2_O_2_) production from oxidation of xanthine oxidase by the substrate xanthine, utilizing the human xanthine oxidase assay kit (Sigma USA). The progress of reaction was observed through thin layer chromatography (TLC) on 0.25 mm precoated silica gel plates purchased from Merck, reaction spots were envisaged in iodine compartment and UV. Melting points were measured using a Sonar melting point apparatus and uncorrected. ^1^H NMR and ^13^C NMR spectra were documented in DMSO and deuterated CDCl_3_ respectively on Bruker Avance II 400 NMR spectrometer at the frequency of 400 MHz using tetramethylsilane standard (downfield) moreover chemical shifts were expressed in ppm (δ) using the residual solvent line as internal standard. Infrared (IR) spectra were recorded on Perkin Elmer FTIR spectrophotometer by utilizing KBr pellets system.

### Molecular docking

In silico docking studies was done with integrated Schrodinger software using Glide module for enzyme ligand docking [32]. The 3D crystal structure of xanthine oxidase co-crystallized with salicylic acid was retrieved from Protein Data Bank (PDB ID. 2E1Q). The 3D-structures of hesperitin derived compounds to be docked against XO were built in maestro building window. Ligand preparation was performed in Ligprep module. The targeted protein structure was further refined in the Protein Preparation Wizard en route to obtain the optimized and chemically accurate protein configuration. For that, the co-crystallized enzyme (XO) was retrieved directly from Protein data bank in maestro panel followed by removal of water molecules, addition of H atoms, addition of missing side chains and finally minimization was done to obtain the optimized structure. Validation of docking protocol was done by redocking the co-crystallized ligand and RMSD value of 1.2 Å was found which is quite satisfactory to approve the protocol. During the last step of docking, the receptor grid generation tool was utilized to find out the active/binding site for docking and glide docking with extra precision was used to visualize the interaction of protein and ligand.

### Synthesis of hesperitin derivatives

**Scheme 1:**
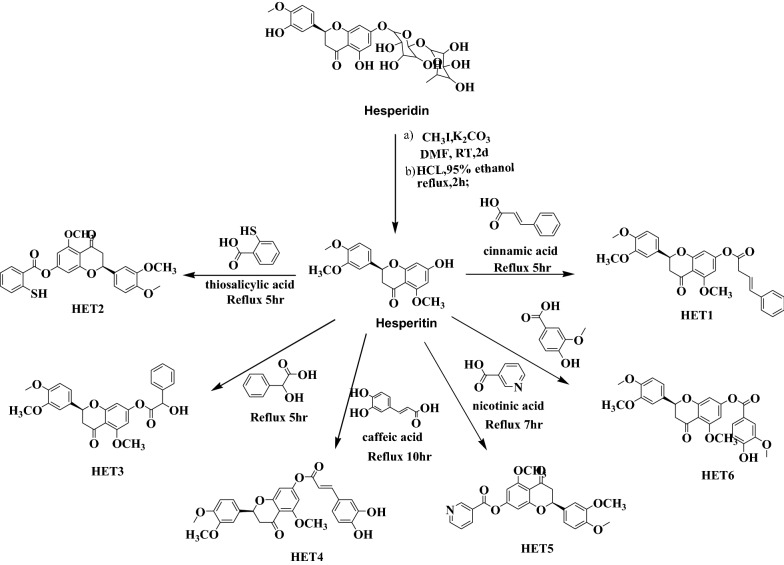
Synthesis of hesperitin derivatives

Derivatives of lead compound hesperitin were selected and will be synthesized by established scheme (Scheme 1). The derivatives were synthesized, and reaction was monitored by TLC and spectral characterization was done by IR, NMR and mass spectroscopy.

#### Procedure for synthesis of hesperitin derivatives

##### General procedure for the synthesis of methylated hesperitin

Hesperidin was first methylated by methyl iodide in presence of potassium carbonate and dimethyl formamide by stirring along with reflux at 40 °C for 48 h to generate methylated hesperidin. Acidolysis of above was done to obtain the methylated hesperitin by refluxing it with HCl and 95% ethanol for 4 h.

##### General procedure for the synthesis of ester derivatives of hesperitin

Hesperitin (0.01 mol) was dissolved in diethyl ether and refluxed with different phenolic (0.01 mol) acid to obtain their ester derivatives.

### Spectral data

HET1: yield 55.6% R_f_ 0.6 [mobile phase for TLC—ethyl acetate:methanol:water (15:3:2)] M.pt. (232–233) IR (KBr pelletets) cm^−1^ 1633 (C=C str.), 1725 (C = 0 str., ester), 1143 (C–O str.), 1021 (O–CH_3_ str.), ^1^H NMR (400 MHz, DMSO-*d*_6_) δ 7.53–7.41 (m, 5H), 7.28 (ddd, *J* = 7.4, 1.5, 0.6 Hz, 1H), 7.10 (dd, *J* = 1.5, 0.6 Hz, 1H), 6.97–6.92 (m, 2H), 6.80 (d, *J* = 1.5 Hz, 1H), 6.56 (dp, *J* = 15.1, 0.7 Hz, 1H), 6.38 (dt, *J* = 15.1, 6.1 Hz, 1H), 5.53 (tt, *J* = 7.0, 0.7 Hz, 1H), 3.89 (d, *J* = 8.4 Hz, 6H), 3.52 (dt, *J* = 6.1, 1.0 Hz, 2H), 3.32 (dd, *J* = 12.4, 7.0 Hz, 1H), 3.24 (dd, *J* = 12.4, 7.0 Hz, 1H), 2.48 (d, *J* = 0.5 Hz, 3H) ^13^C NMR (100 MHz, chloroform-*d*) δ 193.30, 169.55, 162.29, 155.19, 148.49, 148.45, 141.05, 136.54, 131.92, 131.44, 128.51, 126.33, 123.65, 120.02, 117.33, 116.98, 113.81, 112.91, 108.79, 78.52, 56.36, 56.12, 42.67, 38.44, 30.87 m/z found for C_28_H_26_O_6_:458 (M^+^) 459 (M+1)^+^. Anal calcd for C_28_H_26_O_6_: C, 73.35; H, 5.72; O, 20.94 found: C, 73.35; H, 5.75; O, 20.95.

HET2: yield 72.5% R_f_ 0.7 [mobile phase for TLC—ethyl acetate:methanol:water (15:3:2)] M.pt. (225–226) IR (KBr pelletets) cm^−1^ 2524 (S–H str., Ar), 1662 (C=C str.), 1710 (C=0 str., ester), 1160 (C–O str.), 1231 (O–CH_3_ str.), ^1^H NMR (400 MHz, DMSO-*d*_6_) δ 8.07 (dd, *J* = 7.4, 1.6 Hz, 1H), 7.48 (dd, *J* = 7.4, 1.6 Hz, 1H), 7.39 (td, *J* = 7.4, 1.6 Hz, 1H), 7.31 (td, *J* = 7.4, 1.6 Hz, 1H), 7.18 (ddd, *J* = 7.5, 1.5, 0.6 Hz, 1H), 7.13 (dd, *J* = 1.5, 0.6 Hz, 1H), 6.99–6.93 (m, 2H), 6.84 (d, *J* = 1.5 Hz, 1H), 5.52 (tt, *J* = 7.0, 0.6 Hz, 1H), 3.73 (d, *J* = 8.4 Hz, 6H), 3.36 (dd, *J* = 12.4, 7.0 Hz, 1H), 3.08 (dd, *J* = 12.4, 7.0 Hz, 1H), 2.44 (d, *J* = 0.5 Hz, 3H) ^13^C NMR (100 MHz, chloroform-*d*) δ 197.93, 165.65, 161.30, 150.19, 146.85, 141.11, 138.75, 137.97, 134.15, 133.97, 130.14, 126.30, 126.21, 126.21, 116.08, 116.03, 114.52, 114.24, 112.37, 72.20, 58.87, 57.16, 40.47, 22.59 m/z found for C_25_H_22_O_6_S:450 (M^+^) 451 (M+1)^+^. Anal calcd for C_25_H_22_O_6_S: C, 66.65; H, 4.92; O, 21.31; S, 7.12 found: C, 66.65; H, 4.96; O, 21.31; S, 7.15.

HET3: 62.0% R_f_ 05[mobile phase for TLC—ethyl acetate:methanol:water (15:3:2)] M.pt. (220–222) IR (KBr pelletets) cm^−1^3441 (O–H str., Ar), 1668 (C=C str.), 1723 (C=0 str., ester), 1124 (C–O str.), 1100 (O–CH_3_ str.), ^1^H NMR (400 MHz, DMSO-*d*_6_) δ 7.42–7.36 (m, 3H), 7.36–7.27 (m, 2H), 7.21 (ddd, *J* = 7.5, 1.5, 0.6 Hz, 1H), 7.02 (dd, *J* = 1.5, 0.6 Hz, 1H), 6.95–6.86 (m, 2H), 6.80 (d, *J* = 1.5 Hz, 1H), 5.54 (tt, *J* = 7.0, 0.7 Hz, 1H), 5.07 (t, *J* = 0.9 Hz, 1H), 3.82 (d, *J* = 8.4 Hz, 6H), 3.29 (dd, *J* = 12.4, 7.0 Hz, 1H), 3.13 (dd, *J* = 12.4, 7.0 Hz, 1H), 2.43 (d, *J* = 0.5 Hz, 3H) ^13^C NMR (100 MHz, chloroform-*d*) δ 196.13, 171.83, 162.30, 156.46, 148.49, 148.45, 141.08, 136.97, 131.44, 128.67, 127.54, 120.02, 117.34, 117.02, 113.81, 112.91, 108.87, 78.52, 73.35, 55.89, 55.88, 44.61, 27.52 m/z found for C_26_H_24_O_7_:448 (M^+^) 449 (M+1)^+^. Anal calcd for C_26_H_24_O_7_: C, 69.63; H, 5.39; O, 24.97 found: C, 69.66; H, 5.41; O, 24.97.

HET4: 73.3% R_f_ 0.6 [mobile phase for TLC—ethyl acetate:methanol:water (15:3:2)] M.pt. (210–212) IR (KBr pelletets) cm^−1^ 3424 (O–H str., Ar), 1635 (C=N str.), 557 (C–Cl str.), 1147 (C–O–C str.), 1511 (NO_2_ str.), 1021 (O–CH_3_ str.) ^1^H NMR (400 MHz, DMSO-*d*_6_) δ 7.71–7.62 (m, 2H), 7.28 (ddd, *J* = 7.5, 1.5, 0.6 Hz, 2H), 7.08–6.99 (m, 4H), 6.97 (dd, *J* = 1.5, 0.6 Hz, 1H), 6.94–6.88 (m, 5H), 6.83–6.77 (m, 6H), 5.51 (tt, *J* = 7.0, 0.6 Hz, 2H), 3.86 (d, *J* = 8.4 Hz, 12H), 3.27 (dd, *J* = 12.4, 7.0 Hz, 2H), 3.11 (dd, *J* = 12.4, 7.0 Hz, 2H), 2.49 (d, *J* = 0.5 Hz, 6H).^13^C NMR (100 MHz, chloroform-*d*) δ 190.79, 164.32, 161.30, 155.87, 149.64, 149.37, 148.55, 147.02, 145.25, 141.08, 131.44, 126.58, 122.67, 120.02, 117.02, 116.62, 116.36, 115.86, 115.08, 113.81, 112.91, 107.81, 75.91, 54.86, 54.03, 44.61, 21.02 m/z found for C_27_H_24_O_8_:476 (M^+^) 477 (M+1)^+^. Anal calcd for C_27_H_24_O_8_: C, 68.06; H, 5.08; O, 26.86 found: C, 68.09; H, 5.09; O, 26.88.

HET5: 71.5% R_f_ 0.8 [mobile phase for TLC—ethyl acetate:methanol:water (15:3:2)] M.pt. (219–221) IR (KBr pelletets) cm^−1^ 1650 (C=N str.), 1627 (C=C str.), 1730 (C=0 str., ester), 1176 (C–O str.), 1234 (O–CH_3_ str.) ^1^H NMR (400 MHz, DMSO-*d*_6_) δ 9.17 (d, *J* = 1.5 Hz, 1H), 8.74 (ddd, *J* = 7.5, 1.5, 0.4 Hz, 1H), 8.14 (dt, *J* = 7.5, 1.5 Hz, 1H), 7.51 (t, *J* = 7.5 Hz, 1H), 7.23 (ddd, *J* = 7.5, 1.5, 1.6 Hz, 1H), 7.09 (dd, *J* = 1.5, 0.6 Hz, 1H), 6.96–6.94 (m, 2H), 6.81 (d, *J* = 1.5 Hz, 1H), 5.56 (tt, *J* = 7.0, 0.6 Hz, 1H), 3.76 (d, *J* = 8.2 Hz, 6H), 3.37 (dd, *J* = 12.0, 7.0 Hz, 1H), 3.19 (dd, *J* = 12.2, 7.0 Hz, 1H), 2.39 (d, *J* = 7.5 Hz, 3H) ^13^C NMR (100 MHz, chloroform-*d*) δ 197.47, 163.01, 162.30, 155.72, 153.75, 151.34, 146.36, 141.11, 137.45, 131.44, 125.44, 123.43, 120.02, 116.30, 114.86, 114.82 112.37, 104.75, 77.67, 51.86, 51.43, 47.67, 19.82 m/z found for C_24_H_21_NO_6_:419 (M^+^) 420 (M+1)^+^. Anal calcd for C_24_H_21_NO_6_: C, 68.73; H, 5.05; N, 3.34; O, 22.89 found: C, 68.75; H, 5.08; N, 3.36; O, 22.89.

HET6: 88.5% R_f_ 0.6 [mobile phase for TLC—ethyl acetate:methanol:water (15:3:2)] M.pt. (254–255) IR (KBr pelletets) cm^−1^ 3412 (O–H str., Ar), 1637 (C=C str.), 1715 (C=0 str., ester), 1196 (C–O str.), 1230 (O–CH_3_ str.), ^1^H NMR (400 MHz, DMSO-*d*_6_) δ 7.61 (dd, *J* = 7.5, 1.5 Hz, 1H), 7.54 (d, *J* = 1.5 Hz, 1H), 7.25 (ddd, *J* = 7.4, 1.5, 0.6 Hz, 1H), 7.01 (dd, *J* = 1.5, 0.6 Hz, 1H), 6.93–6.91 (m, 2H), 6.85–6.80 (m, 2H), 5.45 (tt, *J* = 7.0, 0.6 Hz, 1H), 3.87 (s, 3H), 3.82 (d, *J* = 8.4 Hz, 6H), 3.30 (dd, *J* = 12.4, 7.0 Hz, 1H), 3.20 (dd, *J* = 12.4, 7.0 Hz, 1H), 2.50 (d, *J* = 0.5 Hz, 3H) ^13^C NMR (100 MHz, chloroform-*d*) δ 186, 25,164.43, 162.94, 155.72, 147.51, 147.42, 143.71, 143.38, 141.11, 131.44, 123.55, 121.16, 120.00, 117.13, 117.04, 113.81, 113.20, 112.91, 106.92, 91.15, 56.00, 55.89, 55.88, 43.31, 35.47. 33.31 m/z found for C_26_H_24_O_8_:464 (M^+^) 465 (M+1)^+^. Anal calcd for C_26_H_24_O_8_: C, 67.23; H, 5.21; O, 27.56 found: C, 67.25; H, 5.23; O, 27.55.

### Evaluation of biological activity

#### In vitro evaluation of xanthine oxidase inhibitory activity

The method opted to evaluate the inhibitory potential of hesperitin derivatives was a modified protocol of Sigma, done by UV-spectrophotometric method by using xanthine oxidase activity assay kit purchased from sigma (MAK078, sigma-aldrich.co, USA). The colorimetric product obtained in the form of hydrogen peroxide generated during the oxidation of XO was determined by a coupled enzyme technique, measured at 570 nm in a 96-well plate, using the plate reader EPOCH™ “MICROPLATE READER (BIOTEK)”. One unit of XO is defined as the amount of enzyme that catalyzes the oxidation of xanthine substrate, yielding 1.0 µmol of uric acid and hydrogen peroxide per minute at 25 °C. Reagents used were 44 µL of xanthine oxidase assay buffer, 2 µL xanthine substrate solution and 2 µL of xanthine oxidase enzyme solution. All the solutions mentioned above were mixed to prepare reaction mixture. The different concentrations of synthesized derivatives having final volume 50 µL were prepared in dimethyl sulfoxide (DMSO) and added to 96 well plate. To each well 50 µL of reaction mix was added and mixed well. After 2–3 min initial measurement was taken. The plates were incubated at 25 °C taking measurements at every 5 min. Allopurinol served as positive control. Absorbance at different time intervals was noted for further statistical analysis.

#### In vitro evaluation of antioxidant activity by DPPH method

The antioxidant potential of hesperitin derivatives was performed by DPPH method evaluated in the form of IC_50_ estimated using the ELISA plate reader EPOCH™ “MICROPLATE READER (BIOTEK)”. This method opted for evaluation of free radical scavenging activity of DPPH was base on modified procedure described by Dhiman et al. [33]. The tested compounds were prepared in methanolic solution and reacted with methanolic solution of DPPH at 37 °C. The reaction mixture was prepared in 96-well plate by adding 50 µL of sample, 50 µL mL of methanol and 50 µL of DPPH solution prepared in 0.1 mM in methanol. The mechanism of action of DPPH assay was based on the fact that DPPH radical get reduced during its reaction with an antioxidant compound and results in changes of color (from deep violet to light yellow). The absorbance was read at 517 nm for 30 min at an interval of 5 min of using ELISA microplate reader. The mixture of methanol (5.0 mL) and tested compounds (0.2 mL) serve as blank. Ascorbic acid served as positive control.

#### Hydrogen peroxide scavenging (H_2_O_2_) assay

To compare and best evaluate the antioxidant potential of newly synthesized hesperitin derivatives, hydrogen peroxide assay was performed by the method described by Patel et al. [34] with some modifications. The solution of H_2_O_2_ (100 mM) was prepared via the adding up different concentrations of synthesized derivatives ranging from 5 to 80 μg/mL to H_2_O_2_ solution (2 mL), prepared in 20 mM phosphate buffer of pH 7.4. Finally, the absorbance of H_2_O_2_ was measured at 230 nm after incubating for 10 min next to a blank reading of phosphate buffer without H_2_O_2_. For every measurement, a fresh reading of blank was taken to carry out background correction. For control sample containing H_2_O_2_ was scanned for absorbance at 230 nm. Results calculated as percentage of hydrogen peroxide inhibition was estimated by the formula [(A_b _− A_t_)/A_0_] × 100, where A_b_ is the absorbance of the control and A_t_ is the absorbance of compounds/standard taken as l-ascorbic acid (5–80 μg/mL) are shown in Table 3.

## Result and discussion

### Chemistry

For the synthesis of target compounds, we followed the route as depicted in Scheme 1. Briefly, the Hesperidin the starting material was condensed with methyl iodide and potassium carbonate to afford hesperitin under acid catalyzed conditions. Then ester derivatives were prepared with different natural phenolic acids by refluxing in methanol. Formation of ester was confirmed by formation of ester C=O linkage between hesperitin and phenolic acids. Other spectral characterization was also found in agreement.

### Molecular docking

To rationalize the structure activity relationship observed in this research and to foreknow the potential interaction of the synthesized compounds with XO, molecular simulation studies were carried out using Schrödinger suite (Schrödinger Release 2018-2, Schrödinger, LLC, New York, NY, 2018). The crystal structure of xanthine oxidase with PDB code 2E1Q was adopted for the docking calculations. Based on the docking score and binding energy calculation, top ranking derivatives were established and compared with the IC_50_ calculated from in vitro activity (Table 1). The consequential output of ligand docking in form of docked confirmation exposed the significant binding and revealed that all the in vitro synthesized hesperitin derivatives screened by in silico method could be well fitted into the active cavity/binding site of xanthine oxidase making potential binding interactions with the amino acid of nearby residues in close proximity of binding site. An exhaustive per-residue interaction between the xanthine oxidase and synthesized hesperitin derivatives was analyzed to reveal the binding patterns in the cavity. However, to concise the discussion illustration only for the top two compounds along with the native structure hesperitin and standard drug allopurinol and the results are summarized in Table 1.

**Table 1:** Comparison of in vitro activity and molecular docking studies

Compound	Docking score	▲G (KJ/mol)	IC_50_ (µM)
HET1	− 10.297	− 61.495	18.98 ± 0.50
HET2	− 9.106	− 48.846	23.15 ± 1.25
HET3	− 10.827	− 53.951	12.91 ± 0.72
HET4	− 13.257	− 77.252	09.09 ± 0.03
HET5	− 12.148	− 59.473	10.76 ± 0.05
HET6	− 13.056	− 69.729	11.70 ± 0.01
Hesperitin	− 6.461	− 35.334	29.25 ± 0.12
Allopurinol	− 3.366	− 17.231	10.41 ± 0.72

**Fig. 2:**
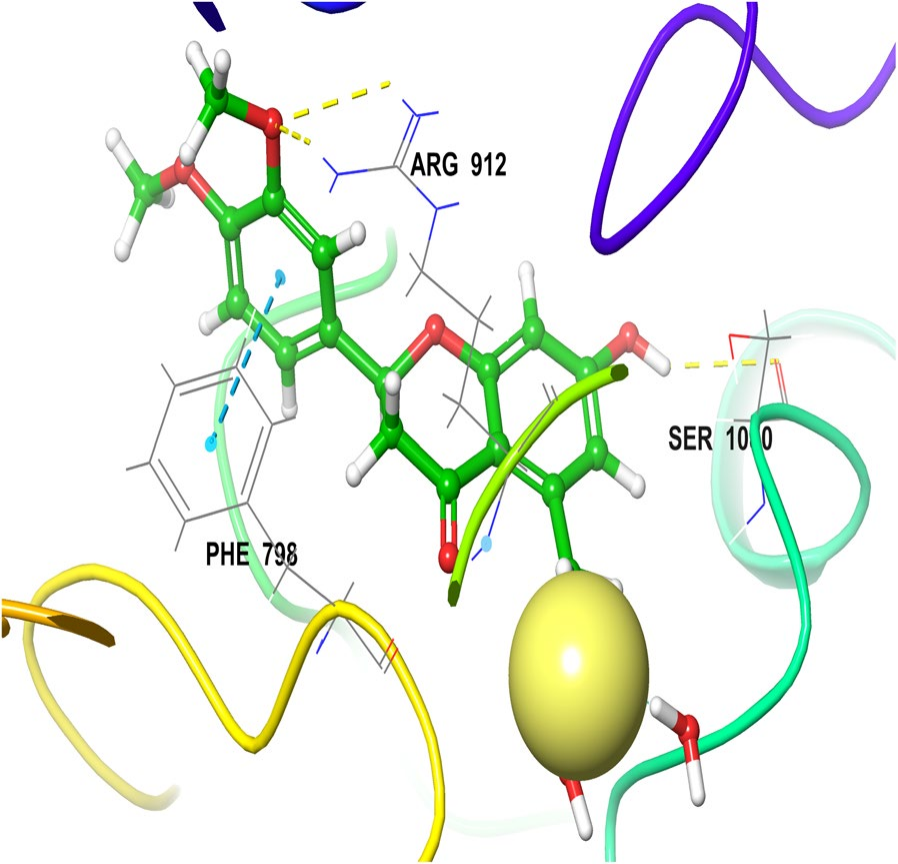
3D view of hesperitin in the active site of xanthine oxidase

Detailed visualization of hesperitin binding poses showed various interactions including hydrophobic, polar and electropositive interactions. The dimethoxy phenyl ring of hesperitin formed a π–π stacking with hydrophobic amino acid PHE798 of XO. This π–π interaction was missing in all the synthesized compounds including most active compound and Allopurinol. From this observation, it could be concluded that pi–pi stacking might be essential for the stability of hesperitin not for the activity. Visual inspection of chroman-4-one moiety of hesperitin elucidates a narrow channel of polar amino acids (GLN767, SER1080, THR1083, GLN1194) surrounded in close proximity of HET4 and forms a H-bond SER 1080 amino acid. Another interesting electropositive interaction was observed between dimethoxy phenyl ring positively charged ARG912 in close vicinity of MOS 1328 (molybdenum atom) which formed a H-bond with GLN767 (Fig. 2).

**Fig. 3:**
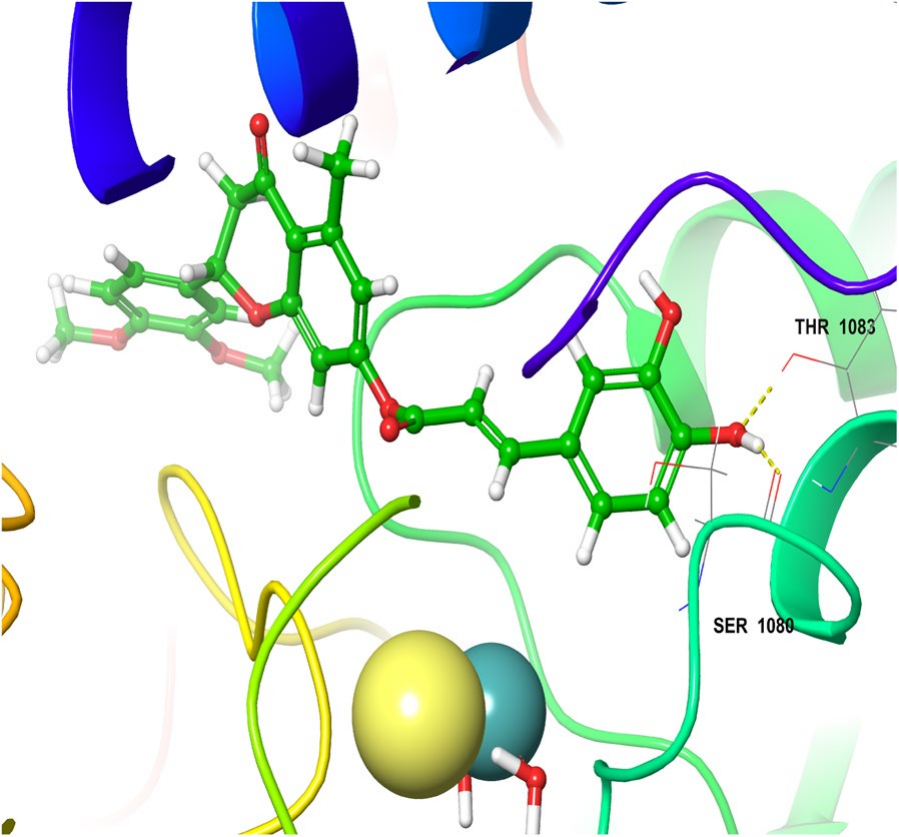
3D view of HET4 in the active site of xanthine oxidase

**Fig. 4:**
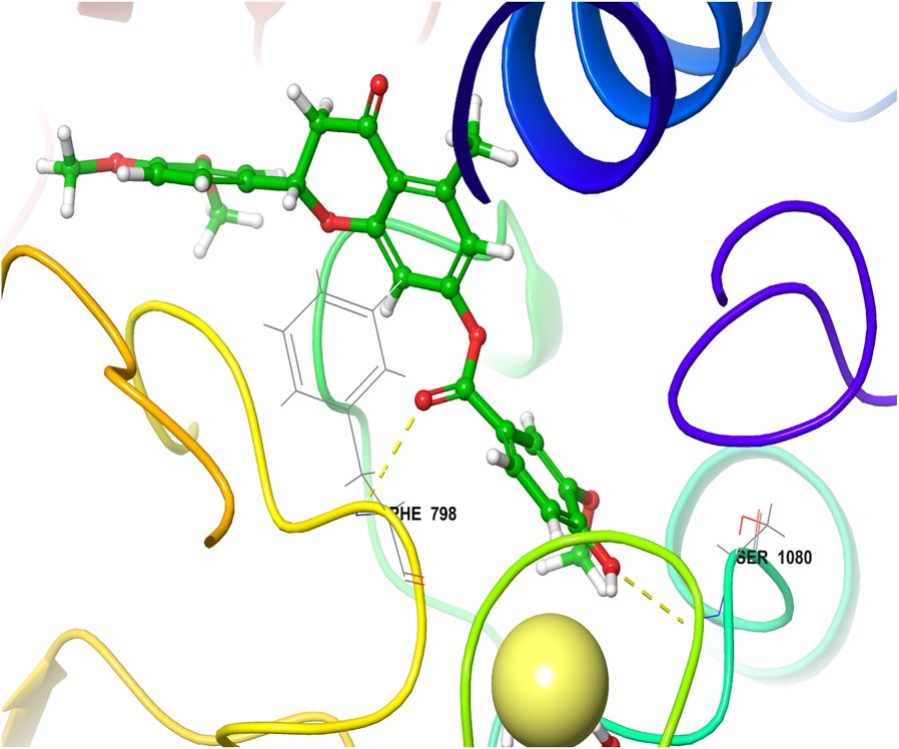
3D view of HET6 in the active site of xanthine oxidase

**Fig. 5:**
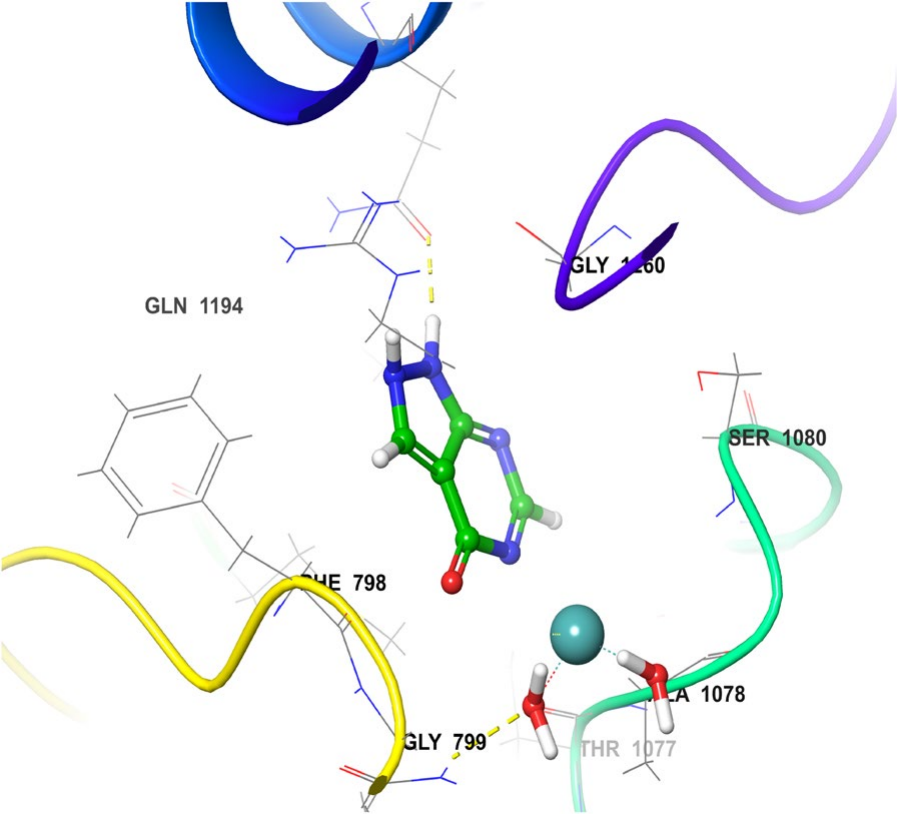
3D view of allopurinol in the active site of xanthine oxidase

The minimized docked conformation of the most active compound HET4 captured in the potentially binding site of XO displayed that HET4 binds at the similar coordinates (Fig. 3) as hesperitin building compact acquaintances with the binding site amino acids by important bonded and non-bonded interactions. The glide score was found to be − 13.257 in comparison to hesperitin (dock score − 6.461) producing an overall binding energy of − 77.252 kcal/mol. The Vander Waals forces contribute maximum share (− 48.709) of binding energy and found to be much established than the electrostatic interactions (− 6.482) when comparing the overall interactive forces of HET4 against XO. In accordance to molecular docking predictions, the dihydroxyphenyl acrylate moiety of HET4 fits within the proteolytic site with good affinity of the xanthine oxidase and is involved, through its hydroxyl oxygen, forming two hydrogen bonds with the polar amino acids SER1080 and THR1083. The oxochroman-7-yl portions, although not forming any direct connections with the neighboring enzyme residues, emerges significant to anchor the centralized part of the ligand defined by the important hydrophobic interactions (ALA1198, PHE798 and MET1038). A very similar binding pattern was exhibited by HET6 (Fig. 4), which retains the inhibitory effect of HET4 possessing a glide score -13.056 and binding energy − 69.729 kcal/mol forming two H-bond with SER1080 and PHE798. The loss of observed activity may be contributed to the missing hydrogen bond in HET6 because of different phenolic acid substitution in HET4. In case of Allopurinol, the active site residues surrounding ligand were almost similar and placed near to MOS 1328. The hydrogen bond was observed between purine ring of Allopurinol and GLN1194 (Fig. 5).

### In vitro xanthine oxidase inhibitory activity

In order to monitor the efficacy of hybridized hesperitin and phenolic acid derivatives, xanthine oxidase inhibitory activity was determined using XO Activity Assay Kit purchased from Sigma-aldrich Co. Allopurinol (positive control) reported to inhibit xanthine oxidase was also analysed in similar environmental conditions to make a comparison with synthesized derivatives. The inhibition ratios revealed the xanthine oxidase inhibitory activity of the synthesized hesperitin derivatives and the results are compiled in Table 1 and compared with results of molecular docking. Phenolic acid derived hesperitin derivatives exhibited remarkable activity comparable to the positive control. All the compounds of this series were effective with IC_50_ values ranging from 09.0 to 23.15 µM. Based on the in vitro activity; it was observed that ester linkage developed from caffeic acid and hesperitin (HET4) comes out most active derivative among all the synthesized compounds showing IC_50_ value 09.094 ± 0.03 µM. Compound HET6 having vanillic acid and HET5 having nicotinic acid substitution also exhibited potential inhibitory activity with an IC_50_ value of 11.702 ± 0.01 µM and 10.769 ± 0.05 µM respectively. All the derivatives showed better activity than hesperitin establishing the importance of ester linkage formed in synthesized derivatives. The results of in vitro activity were found to in concordance of docking studies revealing the significance of in silico screening.

### Enzyme kinetic analysis for XO-inhibitory activity

**Fig. 6:**
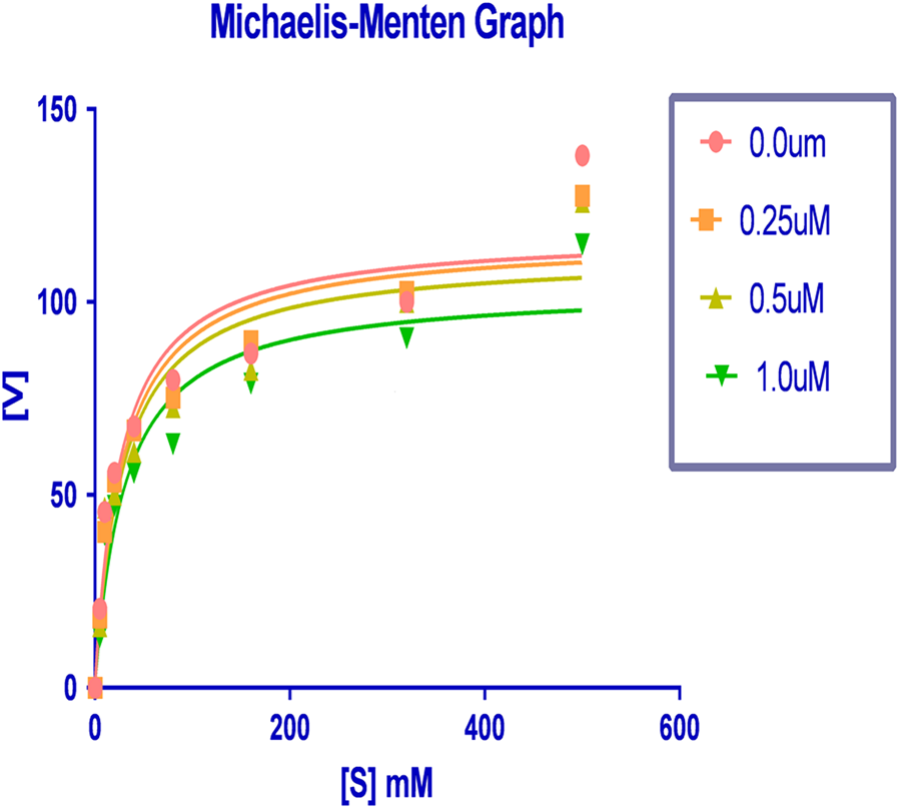
Lineweaver–Burk plot for HET4 against different concentrations

To determine the XO-inhibitory mechanisms of newly synthesized derivatives, we carried out kinetic studies of most active compound HET4 using Graph pad prism software. Firstly Michaelis–Menten curve was plotted for the enzyme activity at different concentrations of HET4 against different concentration of xanthine substrate (Fig. 6).

**Fig. 7:**
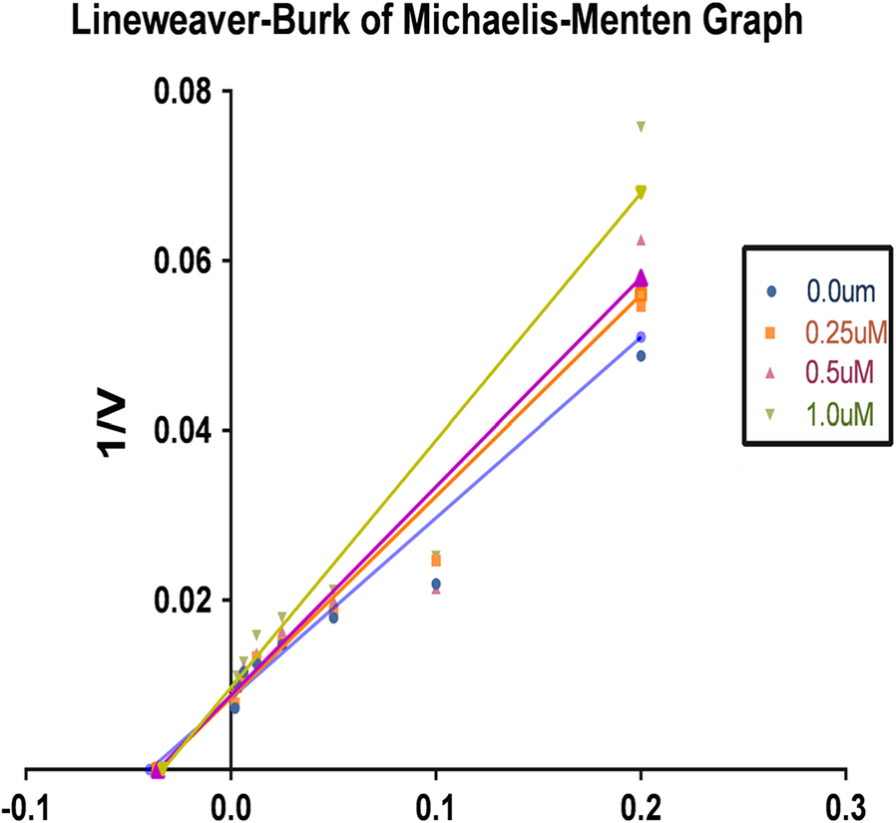
Michaelis–Menten curve for HET4 at different concentrations

Then double reciprocal plot (Lineweaver–Burk) analysis was done in the presence (0.25, 0.5, and 1.0 µM) and absence of HET4 from in vitro data generated during the oxidation of xanthine in presence of xanthine oxidase (Fig. 7). The x- and y-axis intercepts of the Lineweaver–Burk plot were utilized to calculate K_m_ and V_max_ values of HET4 at different concentrations (Table 2).

**Table 2:** K_m_ and V_max_ values of xanthine oxidase at different concentrations of HET4

S. no.	Conc. of HET4 (µM)	K_m_ (µM)	V_max_ (µmol/min)
1	0.0	25.21	117.6
2	0.25	27.91	116.4
3	0.5	28.02	112.2
4	1.0	30.08	103.7

A concentration-dependent decrease of V_max_ was predicted in contrast to K_m_ value which was found to increasing when concentration of HET4 was increased. The intersection of linear straight lines drawn against each concentration was located at same point, suggesting that HET4 reacts in competitive manner during the inhibition of xanthine oxidase.

### In vitro assay for free radical scavenging by DPPH and hydrogen peroxide method

The antioxidant potential of newly synthesized compounds was evaluated by DPPH and Hydrogen peroxide radical assay. The comparative analysis of IC_50_ values for both the assays was done and the results were found to be impressive (Table 3).

**Table 3:** Antioxidant activity of synthesized derivatives by DPPH and H_2_O_2_ assay

Comp.	IC_50_ (µM) ± SEM (DPPH assay)	IC_50_ (µM) ± SEM (hydrogen peroxide assay
HET1	11.117 ± 0.03	12.693 ± 0.41
HET2	06.714 ± 0.15	8.5473 ± 0.06
HET3	0.593 ± 0.25	03.322 ± 0.01
HET4	01.633 ± 0.01	04.642 ± 0.03
HET5	04.821 ± 0.06	06.367 ± 0.26
HET6	07.113 ± 0.13	07.510 ± 0.02
Hesperitin	12.895 ± 0.20	14.425 ± 0.14
Ascorbic acid	22.195 ± 0.08	

*SEM* standard error of the mean

**Fig. 8:**
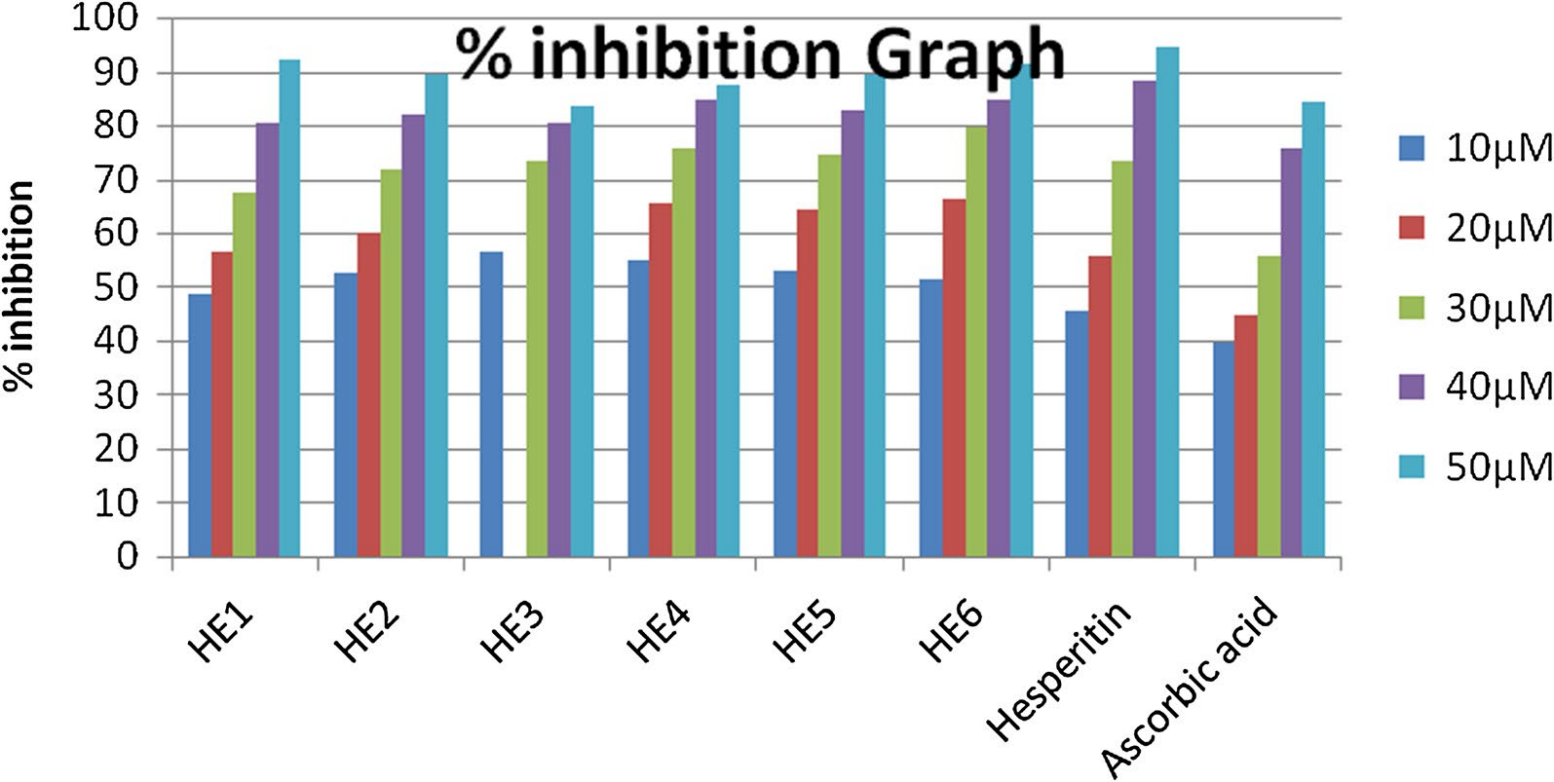
Percentage inhibition graph of synthesized compounds in DPPH assay

The results evinced a noteworthy inhibition of DPPH almost all the compounds when compared with the positive control ascorbic acid (Fig. 8). In case of DPPH assay compound HET4 was demonstrated as most potent compound against oxidative stress caused because of free radicals having an IC_50_ value of 01.633 ± 0.01 µM. Along with this compound HET5 also showed very good antioxidant potential with an IC_50_ value of 04.821 ± 0.06 µM.

**Fig. 9:**
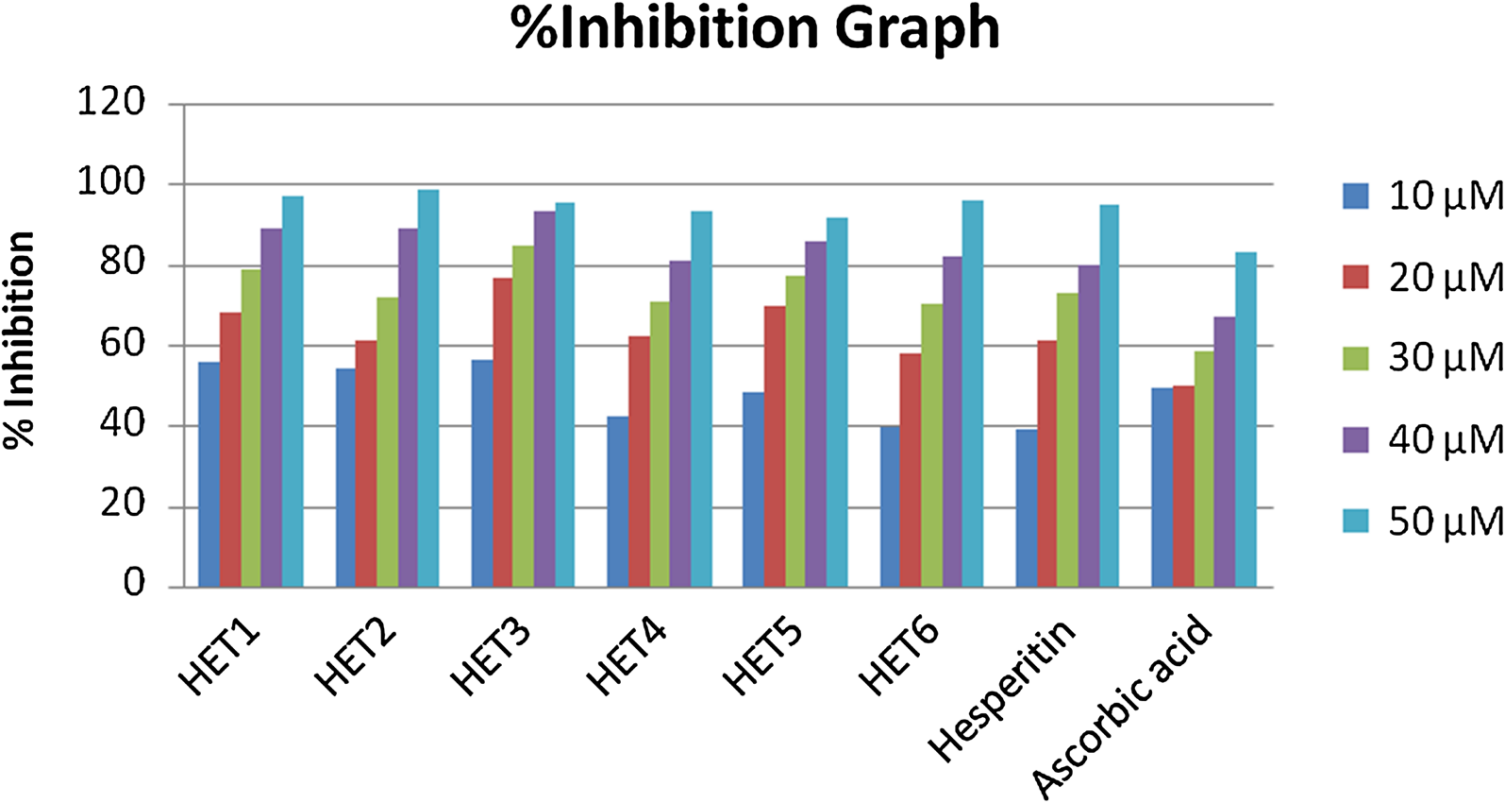
Percentage inhibition graph of synthesized compounds in hydrogen peroxide assay assay

When the detailed structure activity relationship was developed between these compounds, it was concluded that both the compounds have an aromatic OH group attached to the phenyl acrylate moiety. Conversely, during the analysis of hydrogen peroxide assay all the compounds of ester series of hesperitin showed very good antioxidant potential having IC_50_ in range of 03.322 ± 0.01 to 11.117 ± 0.03 (Fig. 9). Compound HET3 having mandelic acid substitution showed potential antioxidant activity among all the derivatives. Along with this caffeic acid substituted HET4 also showed very good scavenging activity with an IC_50_ value of 04.642 ± 0.03.

## Conclusion

Starting from the structures of hesperitin as anti-XO hit previously identified, different hybrid ester of natural phenolic acids was designed and synthesized to explore the structure–activity relationships associated with these xanthine oxidase inhibitors along with their antioxidant potential. Different structural elements were identified as essential for antioxidant and anti-XO properties, such as the presence of OH substituent on aromatic ring (HET4 and HET6), presence of nicotinic ring linker and the overall length and rigidity of the methyl-4-oxochroman-7-yl scaffold. The newly synthesized derivatives with anti-oxidant and ani-XO IC_50_ values in the low micro molar range and good selectivity indexes were identified. Molecular docking provides an improved trail to design the new molecules with an avant-garde stability and potency. This hybridization of two natural constituent could lead to desirable xanthine oxidase inhibitors with improved activity and lower side effects.
